# EcoBank: A flexible database platform for sharing ecological data

**DOI:** 10.3897/BDJ.9.e61866

**Published:** 2021-02-17

**Authors:** Hyun Woo Kim, Sungsoo Yoon, Mokyoung Kim, Manseok Shin, Heenam Yoon, Kidong Kim

**Affiliations:** 1 National Institute of Ecology, Seocheon-gun, South Korea National Institute of Ecology Seocheon-gun South Korea

**Keywords:** EcoBank, ecological database platform, responsive web, WebGIS, stakeholders

## Abstract

**Background:**

Environmental crisis challenges the human race harder than ever before. Ecologists have produced a massive amount of data to cope with the crisis. Accordingly, many national scale ecological database systems have been developed worldwide to manage and analyse these datasets. However, in Korea, ecological datasets produced by different research institutes for different purposes have not been integrated or serviced due to poorly designed information infrastructure. To address this obstacle, we present EcoBank (www.nie-ecobank.kr), an open, web-based ecological database platform designed to play an important role in ecosystem analysis, not only in Korea, but also worldwide.

**New information:**

The architecture of EcoBank comprises core technologies of WebGIS, Application Programming Interface (API), responsive web and open-source software (OSS). Comprehensive ecological datasets from three different sources, including the National Institute of Ecology (NIE) in Korea, related national and international platforms and repositories, enter the three conceptual modules in EcoBank: data management, analysis and service. Diverse potential stakeholders of EcoBank can be classified into three groups: researchers, policy-makers and public users. EcoBank aims to expand its horizons through mutual communication amongst these stakeholders. We opened and launched the EcoBank service in December 2019 and have now begun to broaden its network by linking it to other data platforms and repositories over the globe to find possible solutions to ecological issues in Korea.

## Introduction

One of the ultimate goals of ecological studies is to understand and quantify ecological associations in different spatiotemporal scales (Kissling et al. 2012, Stephens et al. 2019). However, issues in ecology are becoming more challenging due to various negative anthropogenic effects, such as land cover change, global warming and environmental pollution (Araújo and Rahbek 2006, de Chazal and Rounsevell 2009, Huh et al. 2014). It is essential to secure accurate and up-to-date knowledge of the ecosystem to assess these issues.

Ecologists working in different fields have collected data through various methods to address complex environmental problems ([Bibr B6413104][Bibr B6407208], Park 1990, Thuiller 2004). The world of "big data" has arrived in ecology and other branches of the environmental sciences (Farley et al. 2018) with increased quantity, speed and variety of data streams (Hampton et al. 2013). In the field of ecological informatics, various ecological data platforms have been developed worldwide to collect and manage ecological information efficiently (Frehner and Brändli 2006, Michener et al. 2012, Stephens et al. 2019, Belbin and Williams 2016).

Although a few web-based platforms have been designed for managing biodiversity conservation plans and biological resource management in Korea, significant challenges in addressing ecological issues remain. Existing platforms do not use standardised ecological metadata for sharing or organising ecological information. As there is no integrated platform for comprehensive utilisation and one-point access to environmental data, these data are scattered throughout individual institutions, producing particular types of data without consistent format or retrieval capability (Youn 2014). Additionally, many platforms around the world have difficulties in providing user-orientated data due to the lack of open space and systems for quality control (i.e. BES-NET) (Ruckelshaus et al. 2020).

New challenges for managing ecological data require improved interoperability, integration and sharing (Frehner and Brändli 2006). An integrative and comprehensive data platform is strongly recommended in Korea. To settle this need, we present EcoBank in two sections (project description and implementation) to show its extendible framework for exploration, modelling and analysis of distributed ecological data in a web-based environment (Table 1). In the project description section, EcoBank’s developmental phases, core technologies and the conceptual design of EcoBank is described and functions related to exchanging, sharing and analysing spatial data through the internet are discussed. The following implementation section describes functions and services, mainly for target stakeholders. This paper ends with the main conclusions and an outlook for future directions.

## Project description

### Title

EcoBank

### Design description

**development processes, core technologies and their integration**

**Figure 1.**
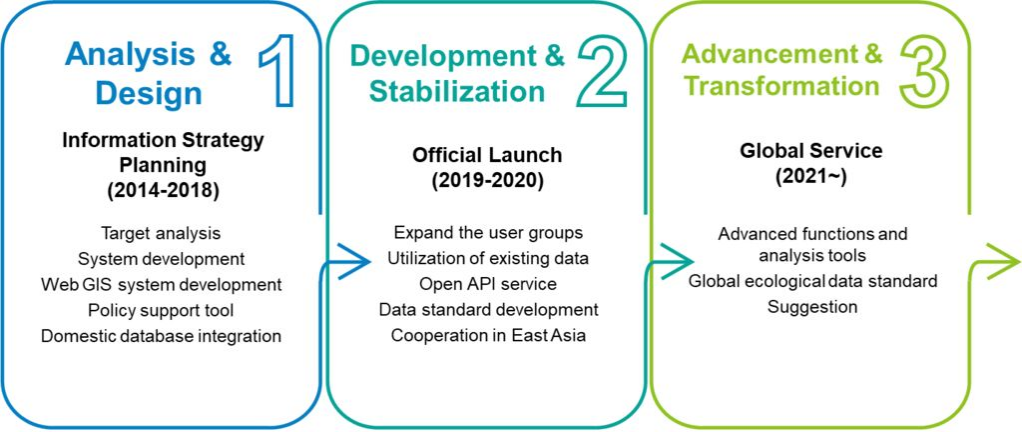
Three phases of EcoBank development processes: Analysis & Design, Development & Stabilizsation and Advancement & Transformation.

**Development processes**: We developed EcoBank through three developmental process phases (Fig. 1). In the first phase (2014-2018), EcoBank development began with information strategy planning, which emphasised long-term management of its data, information and knowledge. Target audience analysis was also conducted with various internal and external expert groups to investigate the opportunities of EcoBank as an international platform in the global market. The main system, including a web geographic information system (WebGIS) and a policy support tool, was developed. Integration of refined and standardised domestic databases into EcoBank began in this phase. In the second phase (2019-2020), EcoBank was officially launched and widely accepted by target stakeholders. The official numbers of accesses were 95,175 in 2019 and 293,300 in 2020. In addition, the numbers of data downloads were 730 in 2019 and 4,388 in 2020. EcoBank provides open API services for application to any data platform. EcoBank has broadened its connections to the East Asian region with various cooperative projects. Additionally, EcoBank developers have suggested an ecological metadata standard, based on the Data Catalog Vocabulary (DCAT). In the third phase (2021~), EcoBank is expected to gain more advanced functions and analysis tools in communication with its stakeholders. International ecological data standards for developing countries in south-eastern Asia, including Vietnam and Thailand, can be derived from its experience and expertise in ecological data management.

**Core technologies**: In this section, core technologies, including WebGIS, Open Source Software (OSS), open API and the responsive web to operate EcoBank properly and efficiently are briefly explained. In the next section, how these technologies are integrated into EcoBank will be discussed.

Research efforts of the geographic information science community have been made to integrate ecological data (Frehner and Brändli 2006). Considering the massive increase in internet users, the traditional GIS paradigm on spatial data handling from a single database has gradually shifted towards a distributed GIS paradigm through physically-distributed database systems or geospatial services (Preston et al. 2003). The technology of web services and open standards has provided the basis for distributed geoprocessing or distributed GIS. A step towards distributed GIS is establishing so-called geoportals that offer gateways to discover and access geographic web services (Maguire and Longley 2005). However, an examination of several existing geoportals has shown that the available functionality is restricted to searching, mapping, publishing and limited querying of distributed geodata (Tait 2005). A missing feature is the availability of comprehensive analysis tools. WebGIS, also known as Web-based GIS or Internet GIS (Peng and Tsou 2003), provides additional means for spatial data analysis as an alternative.

OSS is a type of computer software in which the source code is released under a licence in which the copyright holder grants users the rights to study, change and distribute the software to anyone for any purpose (Lakhani and Von Hippel 2004, Steiniger and Hay 2009).

API is an intermediary medium that helps our services to use features and programmes provided by individual developers, businesses and organisations. If the user interface (UI) connects users and objects (hardware or software) to be handled by users, the API connects the programme to another programme. API is used in almost all ecological data platforms worldwide, such as Virtual Database, DataOne and SPECIES (Frehner and Brändli 2006, Michener et al. 2012, Stephens et al. 2019) and is also used in EcoBank.

Optimising any web-based information for various devices is no longer an option, but a necessity. Due to increasingly diverse smart devices, it is impossible to provide an appropriate service to customers with a desktop-version website alone. Responsive web design is a method to effectively enable the use of various devices (Gardner 2011). EcoBank takes advantage of responsive web design. Layouts are instantly rendered to the best design for many different web contexts, such as mobile phones, tablets and desktops. It not only provides adjustable sizes for webpages, but also optimises components for their own operational principles.

**Figure 2.**
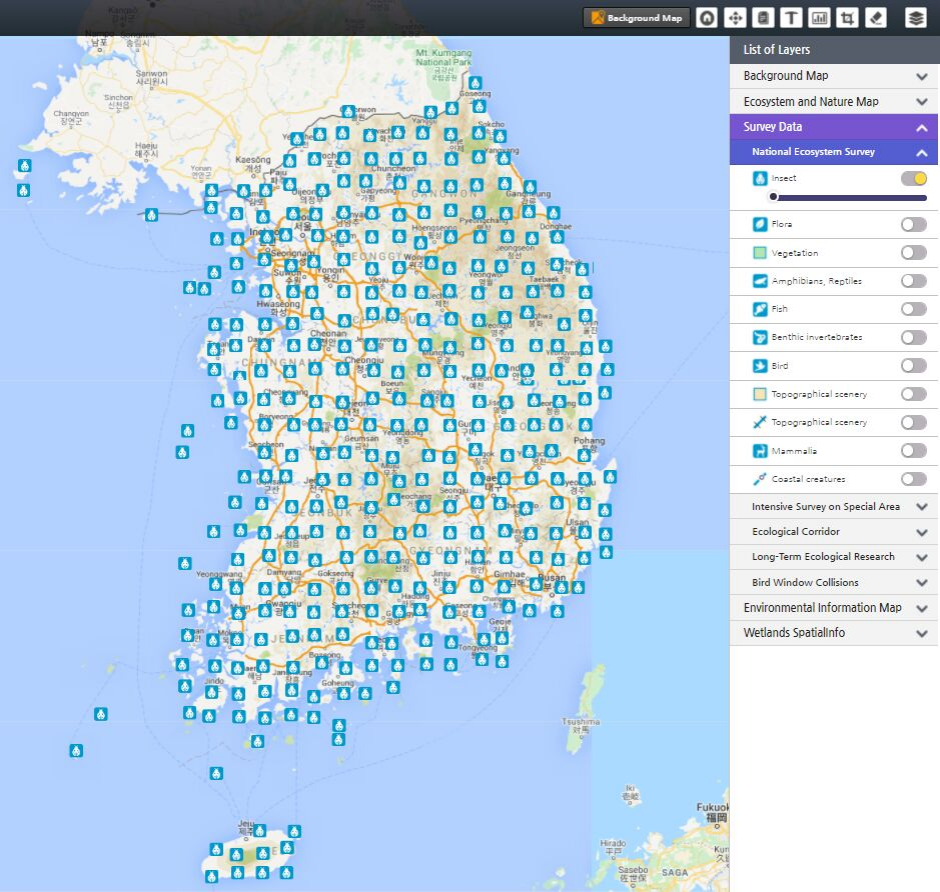
Spatial distributions of insects in South Korea presented by WebGIS in EcoBank. Diverse WebGIS tools, such as Geoserver, OpenLayers, PostGIS and PostgreSQL are successfully integrated into EcoBank.

**Integration of core technologies**: All core technologies were successfully integrated into EcoBank. WebGIS (Fig. 2) allows visualisation of all spatial data (raster and vector) from three input data sources: NIE, other environmental institutes in Korea and international platforms and repositories. WebGIS functions in EcoBank are composed of Geoserver for mapping service, OpenLayers for implementing map service, PostGIS for spatial operation and spatial quarry and PostgreSQL for managing and storing spatial data. The WebGIS solution in EcoBank first collects various ecological information with diverse file formats, such as shape (*.shp), comma-separated values (*.csv) and text-only (*.txt). After that, EcoBank WebGIS loads them into PostgreSQL using PostGIS after diagnosis, transformation and processing to meet the ecological spatial data standard. These loaded spatial data are registered and issued in GeoServer and provided as a map service of the Ecobank using OpenLayers technology. With this distributed GIS, EcoBank intends to have more user-centred functions that will present processed results and provide a chance to overlap data in EcoBank for its own purpose.

**Figure 3.**
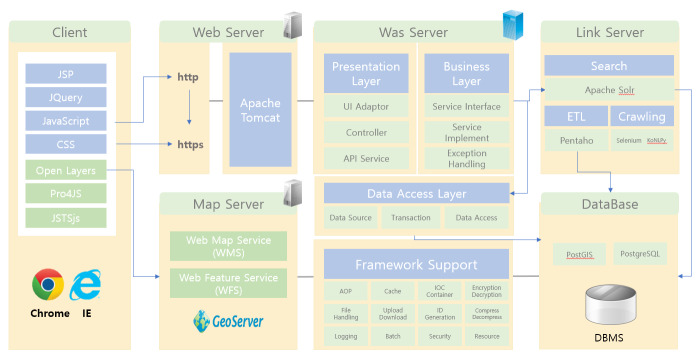
Software packages in EcoBank. All of these packages except AIX (not shown in Figure 3) used in the system architecture of EcoBank, are open-source software.

Almost all software packages (except AIX) applied in EcoBank (Fig. 3) are based on the open-source architecture. For example, Red Hat Enterprise Linux is mounted on a physical web server, a web application server and a linked server. The database server uses PostGIS, PostgreSQL and Selenium, a suite of tools for automating web browsers. KoNLPy, a Python package for processing information in the Korean language, is also applied in EcoBank for web crawling. Other software packages for ETL, Web server and GIS server and Web Client are also OSS packages. EcoBank utilises generalised and OSS packages to eliminate its dependence on software packages of specific companies and observes open standards.

Open API is also efficiently used in connecting and sharing data with other platforms and repositories worldwide. EcoBank provides various ecological spatial information in Web Map Service (WMS) and Web Feature Service (WFS). The WMS service allows one to use map images with diverse formats, such as Portable Network Graphics (PNG), Joint Photographic Experts Group (JPG) and Graphics Interchange Format (GIF) generated from geographic data. The WFS service makes it possible to utilise geographic feature data with vector figures and attributes. To use the OpenAPI of the EcoBank system, an authentication key is needed for each layer to be used. Anyone can ask for an authentification key anytime and it can be used immediately after obtaining approval from the administrator. In addition, EcoBank supports the use of OpenAPI more easily by providing sample codes of programming languages, such as Java, JavaScript, Hypertext Preprocessor (PHP) and Python to use ecological spatial information. For more information, users can visit the OpenAPI in EcoBank (https://www.nie-ecobank.kr/data/api/intrcn.do). Through the API service, EcoBank has already begun linking its data to the largest data repository in Korea’s public sector (Public Data Portal, https://www.data.go.kr/) without any extra processes. Data from EcoBank have been directly transferred and embodied in a different framework. API is a powerful tool for activating EcoBank’s vision of facilitating data use in any other platform in the world. Anyone interested in developing ecological applications using features and datasets in EcoBank can freely utilise the API services of EcoBank.

Responsive web design is also applied to EcoBank. The design concept in the EcoBank website is grouping visual images, infographics and corresponding contents that can be perceived intuitively. The layout of EcoBank’s main page has a vertical grid structure. A content group is deployed according to the access frequency of the user. The EcoBank layout was optimised for 1600 pixels. Meanwhile, the responsive web design leads us to create a UI for accessibility from all devices by focusing on aesthetics and convenience as much as possible.

Based on core technologies briefly explained above, EcoBank eliminates dependencies and complies with open standards utilising open-source based, generalised and open technologies. It guarantees interoperability by providing standards that can be linked to commercial solutions. EcoBank aims for national standardisation. It can be replaced by modularising each service, enabling flexible responses to changes and supporting convenient and diverse environments, such as Eclipse-based modelling, editing, compilation and debugging environments.

**Conceptual Architecture of EcoBank**

**Figure 4.**
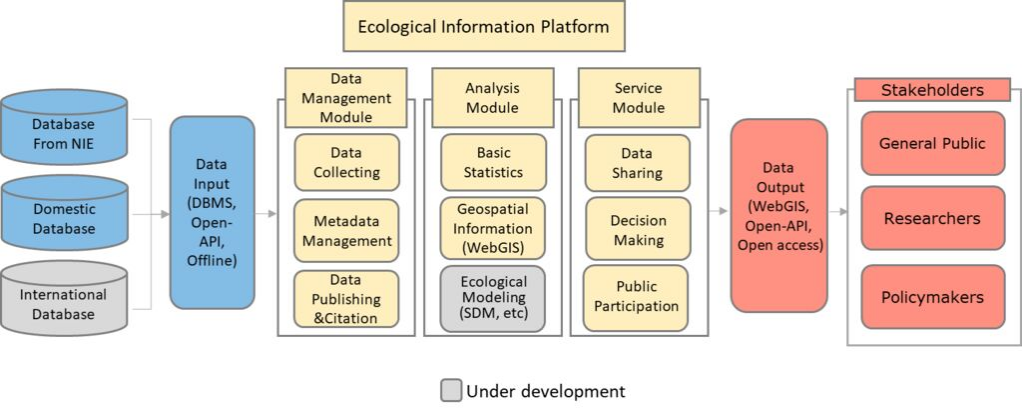
Conceptual architecture in EcoBank: data input resources, ecological information platform and data output.

In this section, the conceptual architecture of EcoBank is presented (Fig. 4). One of the primary goals of EcoBank is to function as the main access point for data and data resources from the National Institute of Ecology (NIE), domestic and international databases (Table 2). NIE itself already has a huge amount of heterogeneous datasets such as the National Ecosystem Survey (NES), the Ecosystem and Nature Map (ENM), the Ecosystem Survey on Special Areas (ESSA), the current status of invasive and ecosystem-disturbing species and results from the National Long-Term Ecological Research projects. The NES, the largest survey project in Korea, has been conducted by the Korean Ministry of Environment for the mainland of South Korea since 1986. ENMs are graded (1–3 grades and separate management areas) for the natural environment, based on ecological and landscape values for mountains, rivers, inland wetlands, lakes, farmlands and cities. The Korean Ministry of Environment commissioned NIE to conduct ESSA to scrutinise biodiversity in national protected areas, such as Ecosystem & Landscape Protection Areas and specific areas including some islands and coastal dunes. These datasets are sent to EcoBank after a rigorous quality control process. We will discuss this in more detail in the data management module section.

EcoBank is exchanging ecological data with other environmental institutes in Korea, such as the National Institute of Biological Research (NIBR), to support the implementation of national policies on biological resources. EcoBank is also linked to a public data portal (https://www.data.go.kr/), which integrates the Korean government’s open data by providing access points on a web-based platform. Thus, the link with PDP can give people more opportunities to access NIE's ecological data. EcoBank is using API services provided by Vworld (https://map.vworld.kr/map/ws3dmap.do), an open platform service on spatial information operated by the Spatial Information Industry Promotion Institute in Korea. Within the territory of Korea, EcoBank can promote GIS services due to higher resolution and more up-to-date spatial information of Vworld maps compared to Google Maps.

EcoBank began to link with international platforms and repositories to boost international stakeholders' participation in finding possible solutions to Korea's ecological issues. Ecological data from Kasetsart University in Thailand and Nong Lam University in Vietnam were stored in EcoBank on a trial basis and other repositories in Asian countries and global data platforms, such as GBIF, will also be connected soon. Data collected from these three routes will be linked to the main server of EcoBank through WebGIS or open API. To handle this diverse and complex stream of incoming data, EcoBank has applied three separate modules. The compositions and purpose of each module are described in the following sections.

**Data management module**: Incoming datasets from the aforementioned three different routes arrive first in a data management module. These datasets need to be organised with standardised metadata for quality control. The quality control processes are performed in three steps. First, we set up the quality diagnosis rules, such as domain rules, range of the dataset, date rules etc. Second, the data qualities are diagnosed, based on the rules established in the first step. Finally, the qualities of the data are improved by correcting errors found in the previous steps.

Due to the absence of any ecological metadata standards in Korea to manage domestic and foreign ecological data in an integrative way, we proposed a metadata schema firstly in Korea to manage and share ecological data with the Korean Telecommunications Technology Association (TTA). Our metadata schema was approved as a national standard of Korea by the TTA in December 2020 (http://www.tta.or.kr/data/ttas_view.jsp?rn=1&pk_num=TTAK.KO-10.1249).

This schema will be useful for efficient data management and interoperability both inside and outside ecological fields.

Digital object identifiers (DOI) enables permanent access, precise identification and reliable citation of data through EcoBank. DOI are currently assigned to datasets produced from a few of NIE’s research projects (Table 2). We will expand the registration of DOI to ecological datasets obtained from GEO DATA (https://geodata.kr/), the first data journal on earth science, ecology, ocean, aerospace and polar research in Korea and research institutes abroad, as well as the remaining NIE research projects.

**Figure 5.**
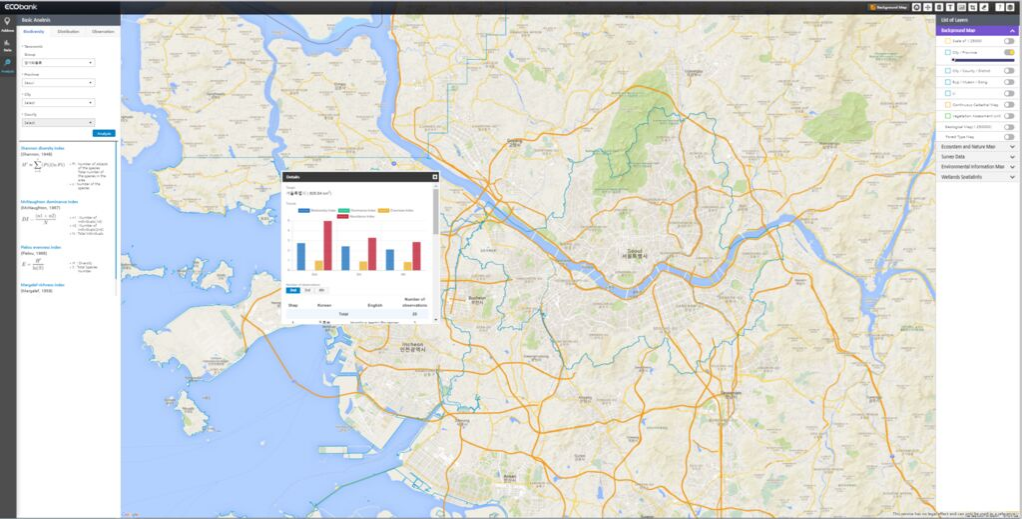
Ecological Indices in Seoul region. EcoBank provides basic analytical services in the Analysis module. Bar plot shows the results of estimating various ecological indices including biodiversity index, dominant index, Evenness Index and Abundance Index on the herpetile species in the Seoul region.

**Analysis module**: The analysis module is implemented within EcoBank with visualised statistical results and Web GIS-based analysis tools. After passing through the quality control process in the management module, datasets are used for analysing biodiversity and modelling species distribution in the Analysis module. Some data produced can be used to evaluate the ENM. Basic ecological analyses on biodiversity and population density can be performed with the collected information (Fig. 5). Researchers can also conduct species distribution modelling (SDM) studies using the MaxEnt machine learning tool built in EcoBank. However, MaxEnt within EcoBank is being tested and will not be fully serviceable until the end of 2021. As an alternative, researchers can carry out SDM with other SDM models, such as the Generalized Additive Model (GAM) and Gap Analysis Program (GAP) after downloading the biodiversity datasets in EcoBank.

**Service module**: EcoBank’s various services for its users are categorised into data-sharing, public participation and policy decision-making support. The data-sharing occurs amongst target users through 'Get Involved', Data & Resources and Open API services in EcoBank. Furthermore, each open dataset that can be identified by a DOI is permanently citable and trackable for users. EcoBank specifies multiple user groups to grant access to data containing sensitive information, such as endangered species, natural reserves and road-kill photos. These layered services meet the needs and usage objectives of various stakeholders in a data platform (Michener et al. 2012). To promote public participation, EcoBank has built discussion boards ('Get Involved' on the menu) for sharing various ideas on ecology and environment and for uploading species observation data. Currently, the NES research project supports ecological expertise to improve the quality of data collected by citizen scientists. Similarly, ecological experts can broaden their ideas or insights regarding ecological issues by discussing with citizens on EcoBank. Policy-makers who have the same interests can plan environmental policies by exchanging ecological information through assigned EcoBank functions. Policy decision support services include opening data produced from environmental policies to the public and getting citizens' feedback. For these purposes, researchers involved in the EcoBank Development project and NES project are actively communicating with citizen scientists through various methods, including educational programmes, emails, phone calls, SNSs etc.

## Web location (URIs)

Homepage: https://www.nie-ecobank.kr/cmmn/Index.do?lang=en

Download page: https://www.nie-ecobank.kr/rsrch/doi/selectDoiRsrchDtaListVw.do

## Technical specification

Interface language: English, Korean

## Usage licence

### Usage licence

Creative Commons Public Domain Waiver (CC-Zero)

## Implementation

### Implements specification

**Figure 6.**
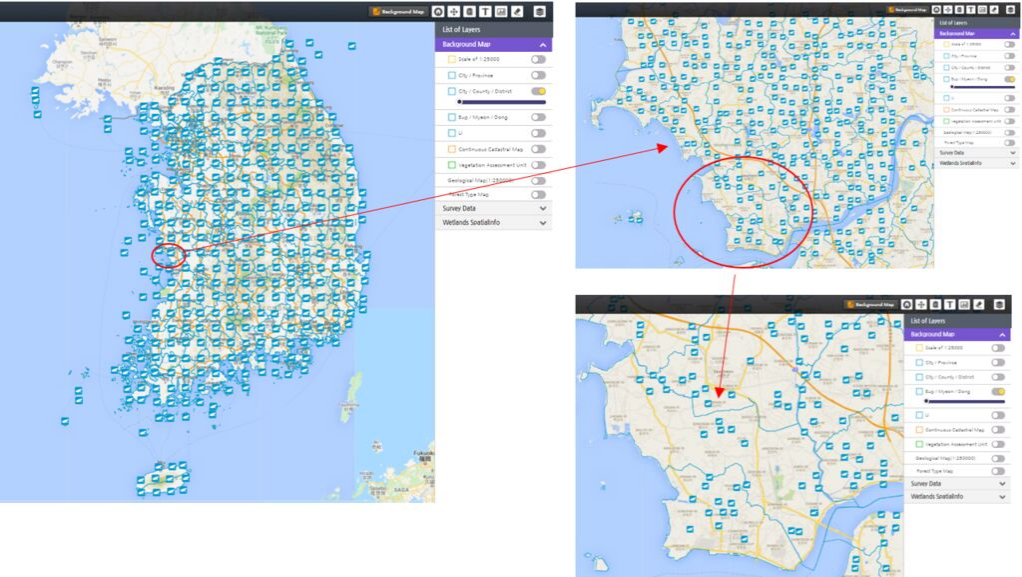
An example of EcoBank Ecospatial Information Service: Distribution of herpetile species in Korea visualised at multiple scales.

**Figure 7.**
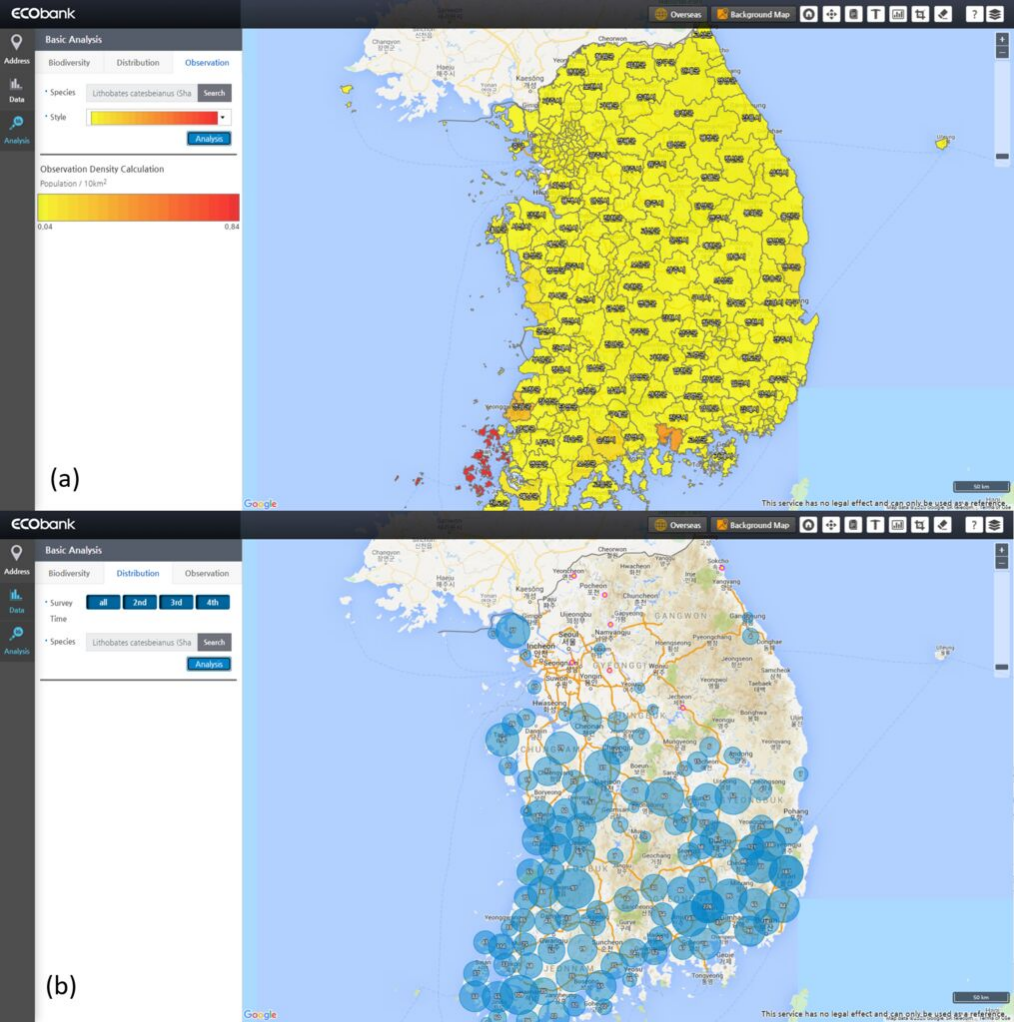
Examples of EcoBank Ecospatial Information Service: Observation density and species distribution of American bullfrog (*Lithobates catesbeianus*) for policy-makers. **a**) Observation density; **b)** Species occurrences.

We present possible types of implementation for each stakeholder. However, these examples are not strictly limited to individual categories, but integrative to each other. For example, researchers and policy-makers have raised the need to study the distribution of herptile (including reptile and amphibian) species at multiple scales regarding ecosystem conservation (Fig. 6). Studies have highlighted that the distribution of herptile species can be the key to develop a universal ecological index (EI) for evaluating the ecological health of aquatic and terrestrial ecosystems (Ali et al. 2018). In addition, research on the detrimental effects of American bullfrogs (Kang et al. 2019) has been addressed by measuring the spatial distribution of this invasive amphibian (Fig. 7). EcoBank is an excellent information source for this purpose, as it provides spatial information for diverse organisms, including herptiles with multi-scale from nationwide to smaller administrative units in Korea.

**Figure 8.**
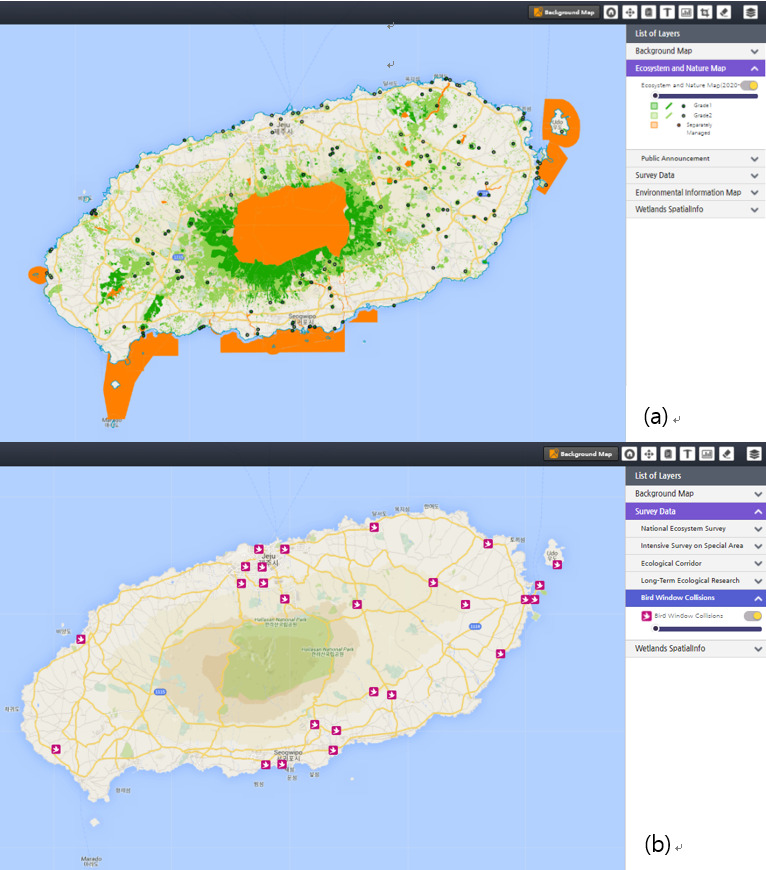
EcoBank for public users. **a)** Ecosystem and Nature Map in Jeju Island; **b)** Citizen-science survey data on bird window collisions in Jeju Island.

With ecotourism in the spotlight recently, Jeju Island, where the tourism industry accounts for more than a quarter of its total economic production, might highly depend on extraordinary ecosystems comprising numerous rare, protected flora and fauna (Kim et al. 2013). ENM gives land use status and improves our general understanding of natural environments in Jeju Island (Fig. 8a). By selecting areas of high ecological value, it is possible to maintain and increase ecosystem services and obtain economic benefits from natural asset use. Therefore, ecological grade information in ENM can be utilised by policy-makers to prepare a land-use plan that regulates excessive developments. Public users will be able to predict and plan for land-use projects where development can be restricted in a similar way (Ahn et al. 2015).

The use of airspace has caused spatial conflict between birds and people. Bird collisions resulting from artificial structures are prime examples of this issue. Thus, researchers need scientific data to understand the status and annual mortality estimates of bird collisions, which has been poorly understood in Korea (Seo 2020). Citizens may collect records of bird collisions from newspapers, verbal communication, online forums and blog posts without any scientific purposes. However, these data retrieved from citizen scientists can be used to publish significant results regarding risk factors and bird collision patterns in scientific journals ([Bibr B6413166][Bibr B6413175]). Although the quality of data produced from citizen scientists has long been a concern due to the lack of quality control processes (Kosmala et al. 2016), Bird Window Collision data have been successfully collected and passed the inner quality control process of EcoBank within a research project through EcoBank (Fig. 8b). With the increasing need for big data, such collaborative projects are expected to deal with ongoing ecological problems.

### Audience

A data platform may sound much superior if it has as many as possible target users as it needs to encompass diverse stakeholder communities. As an example, DataOne (Michener et al. 2012), one of the most successful ecological data platforms, has proposed one primary stakeholder (scientists), five science research environments including academia, government, private industry, non-profit and community and over 20 secondary stakeholders. However, DataONE is well-focused on integrative biological and environmental research, but scientific research is only one of the directions in EcoBank. If we follow a similar approach to DataONE with a wide variety of stakeholders, it may be too complicated and digressive. After reviewing previous studies and consulting with data platform experts, we have realised that it is necessary to set the target users to kick-off a new data platform. We have decided to clarify target users for EcoBank into three groups: researchers, policy-makers and public users. It is possible to include more target users to meet the new needs in the future.

**Researchers**: Professional researchers can find diverse ecological data from EcoBank without paying and asking permission. EcoBank provides the data, not only managed by the National Institute of Ecology, but also other affiliated data producers at one site and presents one with spatial information enabling further studies in many broad dimensions. EcoBank also benefits researchers by offering analysis tools on the website. Researchers can explore and analyse the data and produce results on the same site. The data and the information in EcoBank could be valuable for many researchers.

For example, with species occurrence data, researchers can carry out SDM studies, especially biodiversity changes influenced by climate changes, land-use changes and environmental pollution. The data on ecological statuses can be downloaded and used for environmental impact assessment. It is possible to check the national distribution of species occurrence points and download ecological status data for each species to predict potential species distribution and study habitat suitability. There is also a forum for researchers, "Ecology forum" in the "Get Involved" menu to communicate with other stakeholders including researchers.

**Policy-makers**: Human activities and ecological responses of nature have to include the complex nature of disturbances and stability. Results of such multidimensional interactions can inform policy-makers (Donohue et al. 2016). EcoBank presents ecological data intuitively on the spatial map at multiple scales according to Korean administrative districts so that policy-makers can access important ecological information for their area of interest. Furthermore, the toolbar on the upper right side makes it accessible to overlap other research datasets on the desgnated map for an effcient policy-making process.

Ecological data with high credibility and accessibility are essential in this process. EcoBank provides observation density information on target species to facilitate the establishment of biodiversity conservation areas and develop processes to help national environmental planning. EcoBank's biodiversity-related indices for environmental planning include the Shannon-Wiener Biodiversity Index (Pielou 1969), Dominance Index (McNaughton 1967), Uniformity Index (Pielou 1975) and Abundance Index (Margalef 1958). For more information on biodiversity-related indices in EcoBank, see another article (Sung et al. 2018).

**Public users**: Public users of EcoBank can be divided into three groups: students, business owners and citizen scientists. EcoBank is applicable as educational material. The curriculum for young students in Korea includes environmental education. However, few online materials have been developed for this subject. EcoBank allows free access to all ecological data that can be utilised for any purpose. For example, teachers can develop an educational programme using ecological data to investigate the environmental impact of human behaviour. Conversely, students can generate creative ideas from data simulations in EcoBank.

For business owners, ecological issues and interests have led to the formation of a commodity market in the ecological information field. The related industry, such as ecotourism, has been largely expanded. However, without an efficient sharing and management of ecological data and information, time and resources are wasted constantly. EcoBank can benefit business owners by saving time and money to access data that they need through ENM.

EcoBank can promote citizen science within its space, “Get Involved”. EcoBank also provides a stable and reliable repository for citizen scientists because they can upload ecological survey data with high quality standards under the guidance of professional researchers at NIE. Through this process, the data collected by citizen scientists are established in the NES database. Citizen scientists have recorded their observations of the natural world, including species distribution, phenology and climate data, for centuries (Miller-Rushing et al. 2012). As ecological research has grown into a relatively new area of expertise, the contribution of citizen scientists to ecology is obviously apparent in history, but easily overlooked. Researchers are currently reviewing numerous datasets collected by non-experts to identify long-term changes in the ecosystem. Citizen science in Korea has remained very limited with a relatively low level of civic participation compared to that in European countries and the U.S. (Park 2018). On the other hand, EcoBank already cooperates with amateur scientists in collecting ecological data by providing a user friendly web-based interface. Citizen scientists can be more actively involved in other research projects in NIE, which require up-to-date information, such as invasive species survey and endangered species habitat studies.

## Figures and Tables

**Table 1.** Comparison of EcoBank with diverse other repositories from around the world. EcoBank does not contain datasets on marine biodiversity and does not cover wildlife trades and agricultural information. Except for those, however, EcoBank eventually provides all ecological datasets on terrestrial and freshwater systems in Korea. ^*1^ GBIF: Global Biodiversity Information Facility (https://www.gbif.org/) ^*2^ CCAMLR: Commission for the Conservation of Antarctic Marine Living Resources (https://www.ccamlr.org/en/data/data/) ^*3^ TRAFFIC: The Wildlife Trade Monitoring Network (https://www.traffic.org/) ^*4^ IPBES: Intergovernmental Science-Policy Platform on Biodiversity and Ecosystem Services (https://www.ipbes.net/about)

**Main** **Resources**	**EcoBank**	**Terrestrial Biodiversity** **portals and repositories**	**Marine biodiversity** **portals and repositories**	**Wildlife** **trade** **monitoring** **network**	**IPBES** **portal**
**Examples**	-	GBIF^*1^	CCAMLR^*2^	TRAFFIC^*3^	IPBES^*4^
**Areas**					
Terrestrial	○	○		○	○
Freshwater	○	○			○
Marine		○	○	○	○
**Themes**					
Biodiversity	○	○	○		○
Ecosystems	○	○	○		○
Endangered species	○			○	
Protected areas	○				
Wildlife trade				○	
Climate-related	○	○			
Agriculture		○			
Forest	○				
Indicators	○				
Metrics	○	○			
Standards	○				
Metadata	○				
Taxonomic literature	○				
Analysis	○				
Partner network	○	○	○	○	○

**Table 2.** An overview of structured data in EcoBank. There are four types of data status: data stored in a database server (DB), data implemented through a link with DB servers or API of other data platforms (Link), data implemented through a GIS server (GIS) and data opened in a test server (Test). Databases, which have datasets currently opened with assigned DOI, are in bold type. Data format and size indicate that the format and size of raw data are stored in databases of EcoBank. Data opened only in a test server and linked by API sevices were excluded from the size measurement.

Country	Database	Data status	Data Format	Data Size (MB)	Producer	Update Period
South Korea	**National Ecosystem Survey** ^ab^	DB	SHP	7,316	National Institute of Ecology	1 year
	Ecosystem Survey on Special Areas^a^	DB	SHP	128		1 year
	**Ecosystem and Nature Map** ^ab^	DB	SHP	33,772		1 year
	**Bird Window Collision** ^a^	DB	SHP	8		1 year
	**Road Kill Data** ^ab^	DB	SHP	71		1 year
	Wetland Survey Data^a^	DB	SHP	199		1 year
	Alien Species Data^b^	DB	SHP	1		1 week
	Ecological Corridor Data^ab^	Link (DB)	SHP	3		1 week
	Long-Term Ecological Research^ab^	Link (DB)	SHP	1,130		1 week
	Species List Data^b^	DB	ASCII	303	National Institute of Biological Resources	1 year
	Environmental Conservation Value Assessment Map^a^	DB	ASCII	112	Korea Environmental Institute	Irregular
	Environmental Impact Assessment Information^a^	Link (API)	-	-		Real time
	Soil Phase Symbol^a^	DB	XML	1	National Academy of Agricultural Sciences	Irregular
	Land Cover^a^	DB	SHP	32,453	Ministry of Environment	Irregular
	Forest Type Map^a^	DB	SHP	25,000	Korea Forest Service	Irregular
	DEM^a^	GIS	ASCII	29,000	National Geographic Information Institute	Irregular
	Vworld^a^	Link (API)	-	-	Spatial Information Industry Promotion Institute	Real time
	Serial Cadastral Map^a^	Link (API)	-	-	Ministry of Land, Infrastructure and Transport	Real time
	Local Weather Forecast Information System^b^	Link (API)	-	-	Korea Meteorological Administration	Real time
Thailand	Tree Distribution Data^a^	DB	SHP	-	Kasetsart University (Thailand)	-
Vietnam	Street Tree Data^a^	DB	SHP	-		-
	Land Cover^a^	DB	SHP	-	Nong Lam University (Vietnam)	-
a: data are provided as WMS and WFS services b: data are provided in other types of web services including descriptive statistics and multimedia files						
